# Supplementary figures and images for: Proteasomal inhibition triggers viral oncoprotein degradation via autophagy-lysosomal pathway
Source: PLoS Pathog. 2020 Feb 24;16(2):e1008105. doi: 10.1371/journal.ppat.1008105 (PMC7058366; doi:10.1371/journal.ppat.1008105)

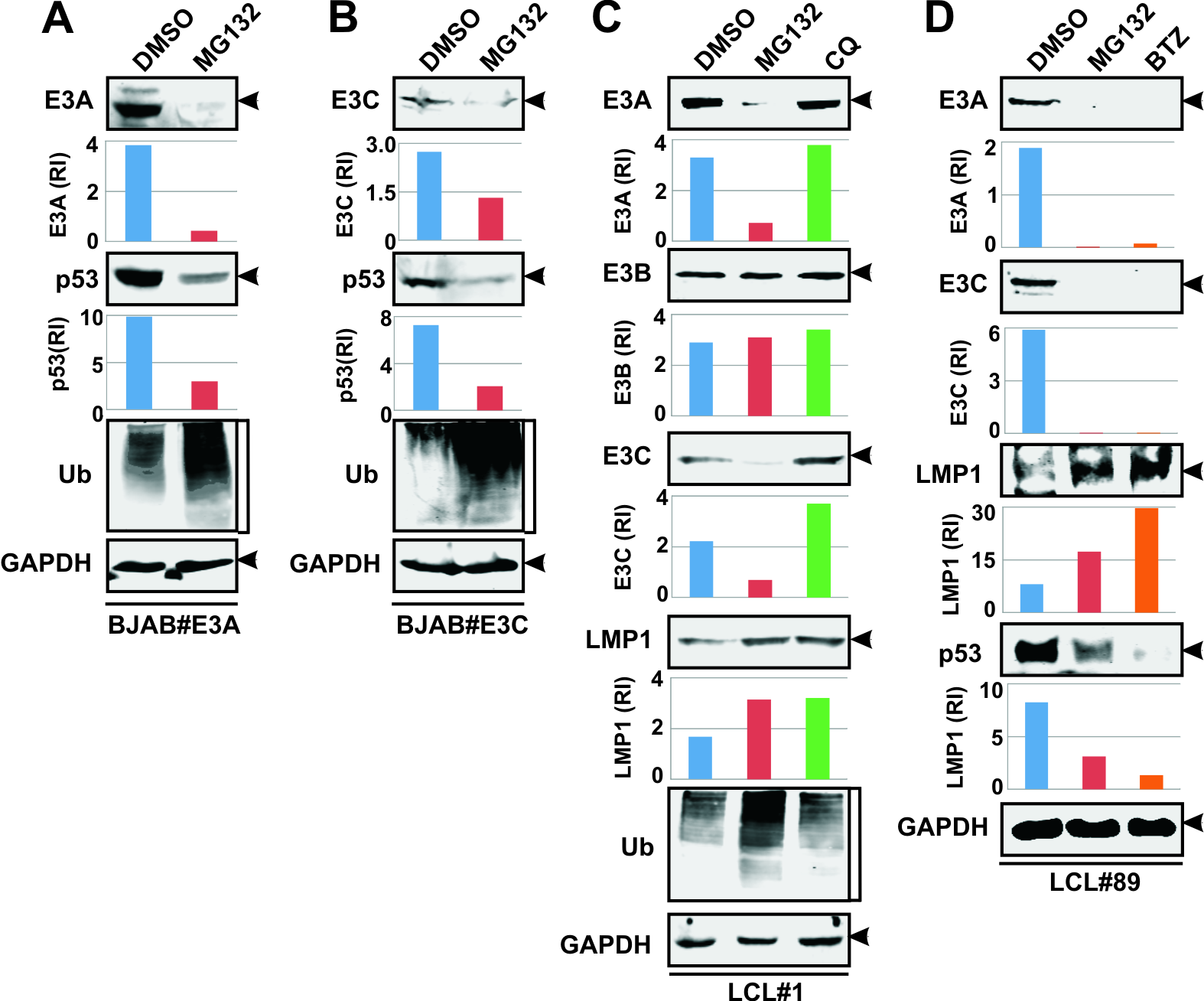

Supplement: S1 Fig — ~10 x 106 (A) BJAB stably expressing EBNA3A (BJAB#E3A), (B) BJAB stably expressing EBNA3C (BJAB#E3C), (C) LCL#1, (D) LCL#89 cells either left untreated (DMSO control) or treated with (A-D) 1 μM MG132, (C) 50 μM chloroquine, CQ (D) 0.5 μM bortezomib for 12 h, were harvested and subjected for western blot analyses using the indicated antibodies. GAPDH blot was used as loading control. Protein bands were quantified by Odyssey imager software and indicated as bar diagrams at the bottom of corresponding lanes. (TIF) [file ppat.1008105.s001.TIF]

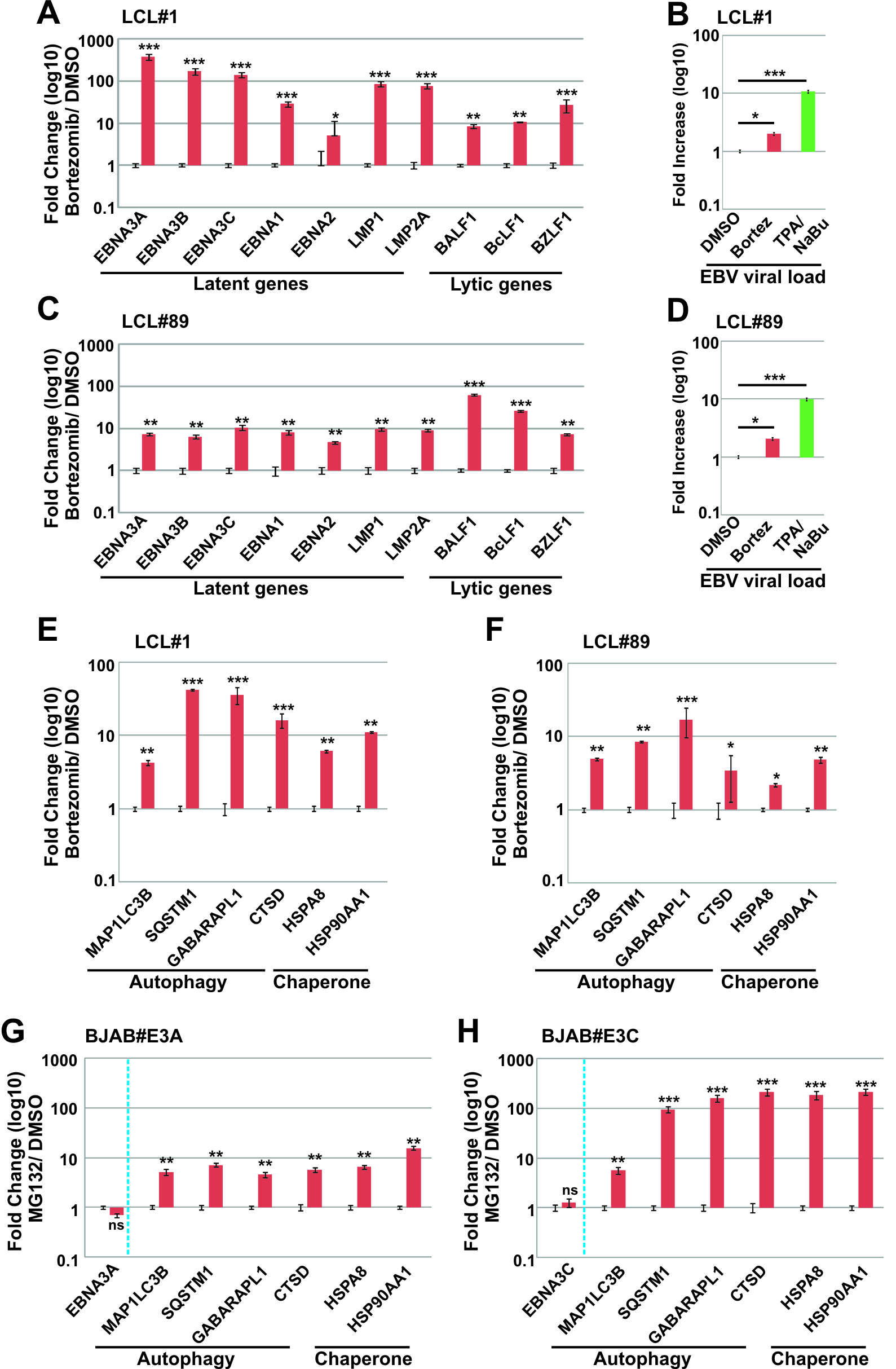

Supplement: S2 Fig — (A-D) ~10 x 106 two LCL clones–LCL#1 and LCL#89 either left untreated (DMSO control) or treated with 0.5 μM bortezomib. 12 h post-treatment cells were harvested for (A and C) total RNA or (B and D) genomic DNA isolation as described in Fig 3. (B and D) LCLs were treated with 3 mM sodium butyrate (NaBu) in combination with 20 ng/ml 12-O-tetradecanoylphorbol-13-acetate (TPA) for 24 h to induce viral lytic cycle as positive control. (A and C) Total RNA was subjected to cDNA preparation followed by qPCR analyses for the selected viral genes. (B and D) qPCR was performed for the detection of EBV DNA (BamHW fragment) using the genomic DNA isolated from each sample. The average fold increase of two independent experiments represented as bar diagrams was calculated in comparison to DMSO control using the 2−ΔΔCt method taking GAPDH as genomic control. (E-F) qPCR analyses of the selected cellular genes as described in (A and C). (G-H) BJAB cells stably expressing (G) EBNA3A (BJAB#E3A) or (H) EBNA3C (BJAB#E3C) either left untreated (DMSO control) or treated with 1 μM MG132 for 12 h were harvested. Total RNA was subjected to cDNA preparation followed by qPCR analyses for the selected viral and cellular gene expressions. (A, C, E-H) For all qPCR analyses, the relative changes in transcripts (log10) using the 2−ΔΔCt method are represented as bar diagrams in comparison to DMSO control using GAPDH and B2M as housekeeping genes. Two independent experiments were carried out in similar settings and results represent as an average value for each transcript. Average values +/- SEM are plotted. *, **, *** = p-value < 0.01, 0.005 and 0.001 respectively. (TIF) [file ppat.1008105.s002.TIF]

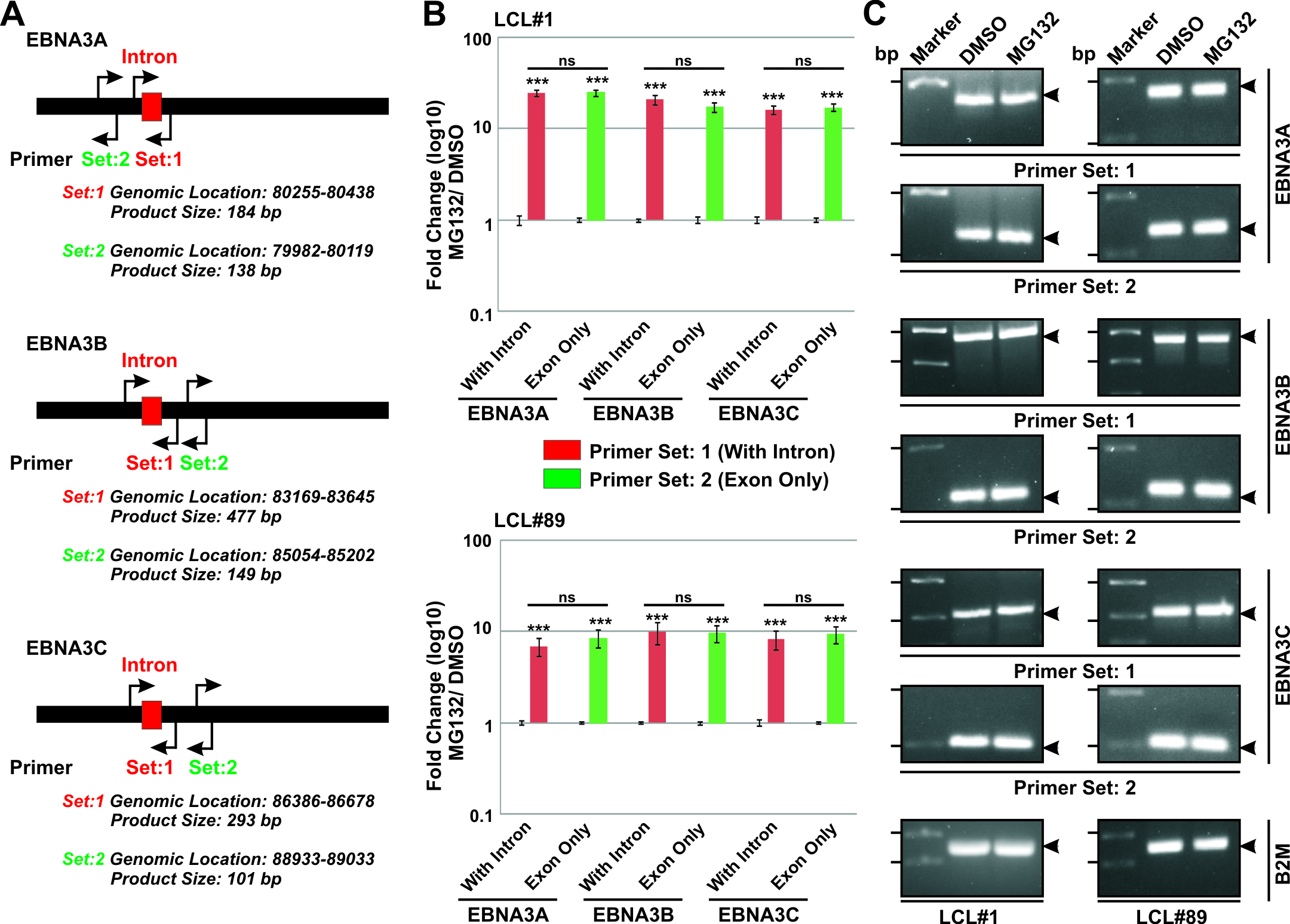

Supplement: S3 Fig — (A) The gene structure of the EBNA3 family (EBNA3A, EBNA3B and EBNA3C) is illustrated, and the names and positions of primers are indicated. Introns and exons are indicated in red and black, respectively. The diagram is not drawn to scale. While primers set 1 indicated as red amplifies intronic region, primers set 2 indicated as green amplifies exclusively exonic region. (B-C) ~10 x 106 two LCL clones–LCL#1 and LCL#89 either left untreated (DMSO control) or treated with 1 μM MG132 for 12 h were harvested for total RNA isolation and subjected to cDNA preparation followed by qPCR analyses for EBNA3 family genes using both primer sets. (B) The relative changes in transcripts (log10) using the 2−ΔΔCt method are represented as bar diagrams in comparison to DMSO control using GAPDH and B2M as housekeeping genes. Two independent experiments were carried out in similar settings and results represent as an average value for each transcript. Average values +/- SEM are plotted. *** = p-value < 0.001 respectively. (C) Agarose gel electrophoresis of end product of each PCR reaction. (TIF) [file ppat.1008105.s003.TIF]

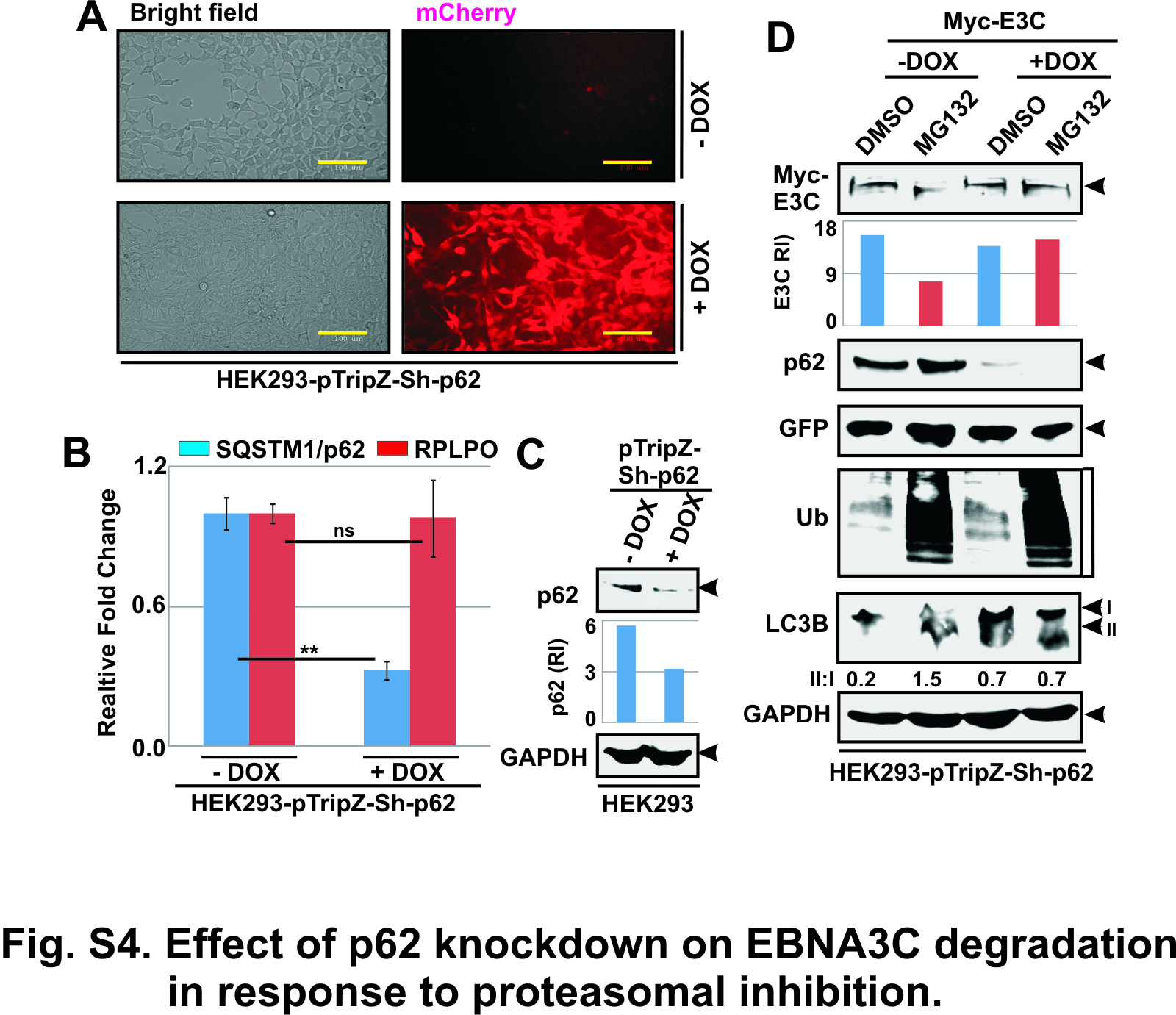

Supplement: S4 Fig — (A) HEK293 cells stably transfected with pTripz-mCherry-Sh-p62 construct expressing sh-p62 under doxycycline (Dox) inducible promoter was treated with 1 μg/ml doxycycline for 48 h and photographed using a fluorescent cell imager. Scale bars, 100 μm. (B-C) 48 h post-treatment, efficiency of p62 knockdown was tested using (B) qRT-PCR and (C) western blot analyses. For qRT-PCR analyses, the relative changes in transcripts using the 2−ΔΔCt method are represented as bar diagrams in comparison to no DOX control using B2M as housekeeping gene. (D) Cells were further transfected either empty vector (pA3M) or myc-tagged EBNA3C expressing construct. 36 h post-transfection cells were either left untreated or treated with 20 μM MG132. 4 h post-treatment cells were harvested, washed with 1 x PBS, lysed in RIPA buffer and subjected for western blot analyses for the indicated antibodies. For western blot analyses, GAPDH blot was used as loading control. Protein bands were quantified by Odyssey imager software and represented as bar diagrams at the bottom of corresponding lanes. (TIF) [file ppat.1008105.s004.TIF]

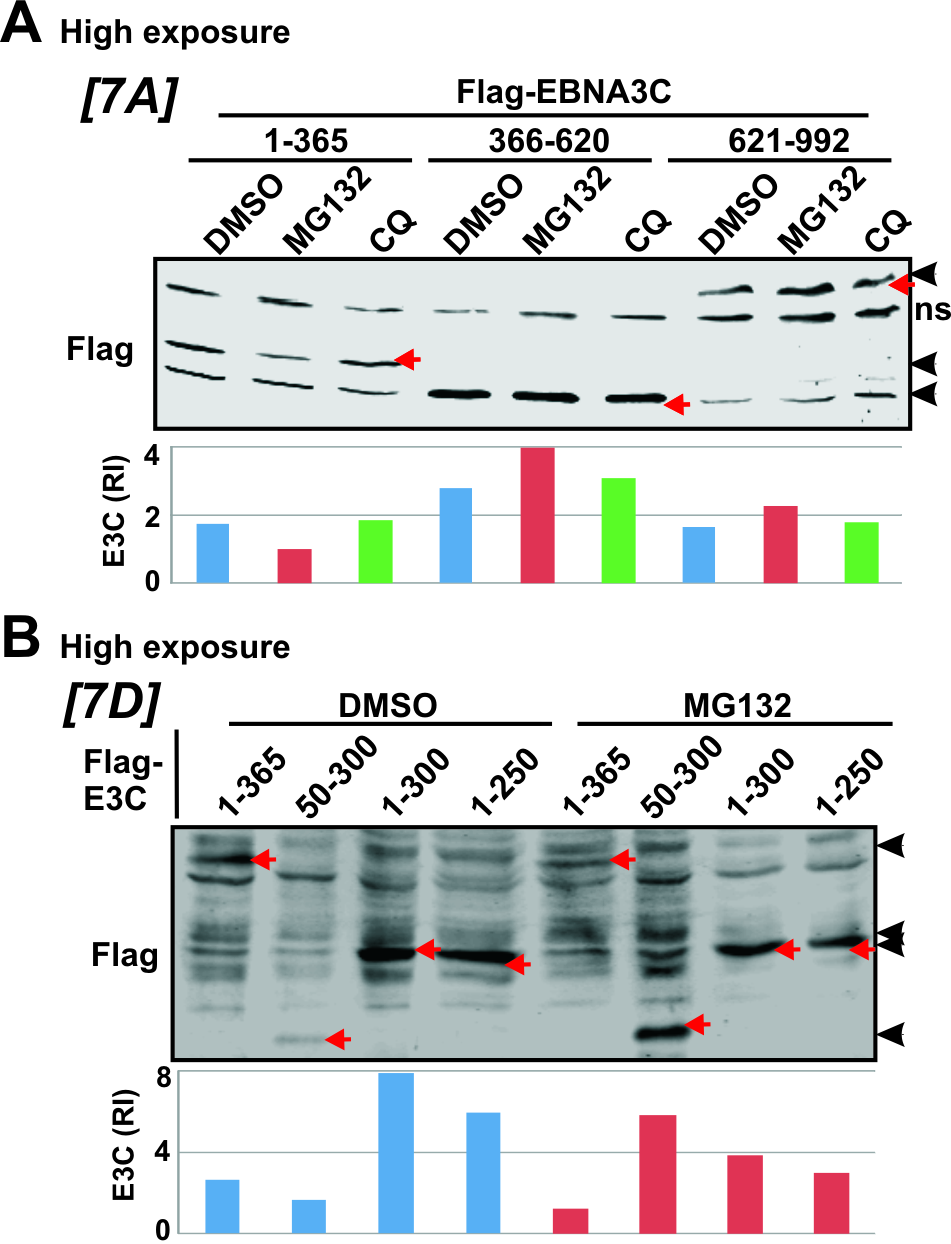

Supplement: S5 Fig — For a better view of protein bands in Fig 7A and 7D, intensities are increased (~3 fold) using Odyssey imager software. (TIF) [file ppat.1008105.s005.TIF]

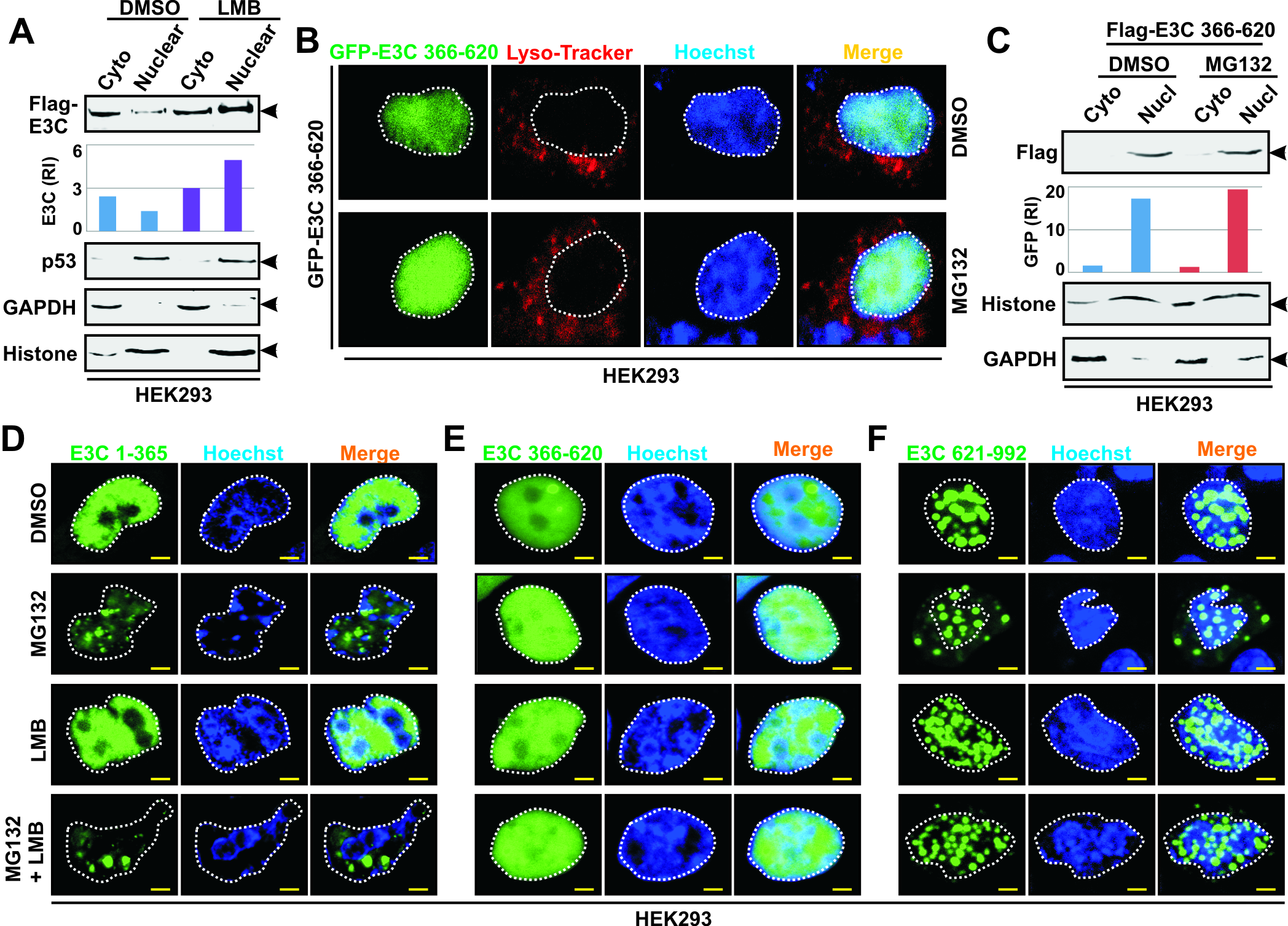

Supplement: S6 Fig — HEK293 cells transiently transfected with flag-tagged EBNA3C construct either left untreated (DMSO control) or treated with leptomycin B (LMB; 20 ng/ml) for 24 h, were subjected to subcellular fractionation as described in the “Materials and Methods” section. Protein bands were quantified by Odyssey imager software and indicated as bar diagrams at the bottom of corresponding lanes. (TIF) [file ppat.1008105.s006.TIF]

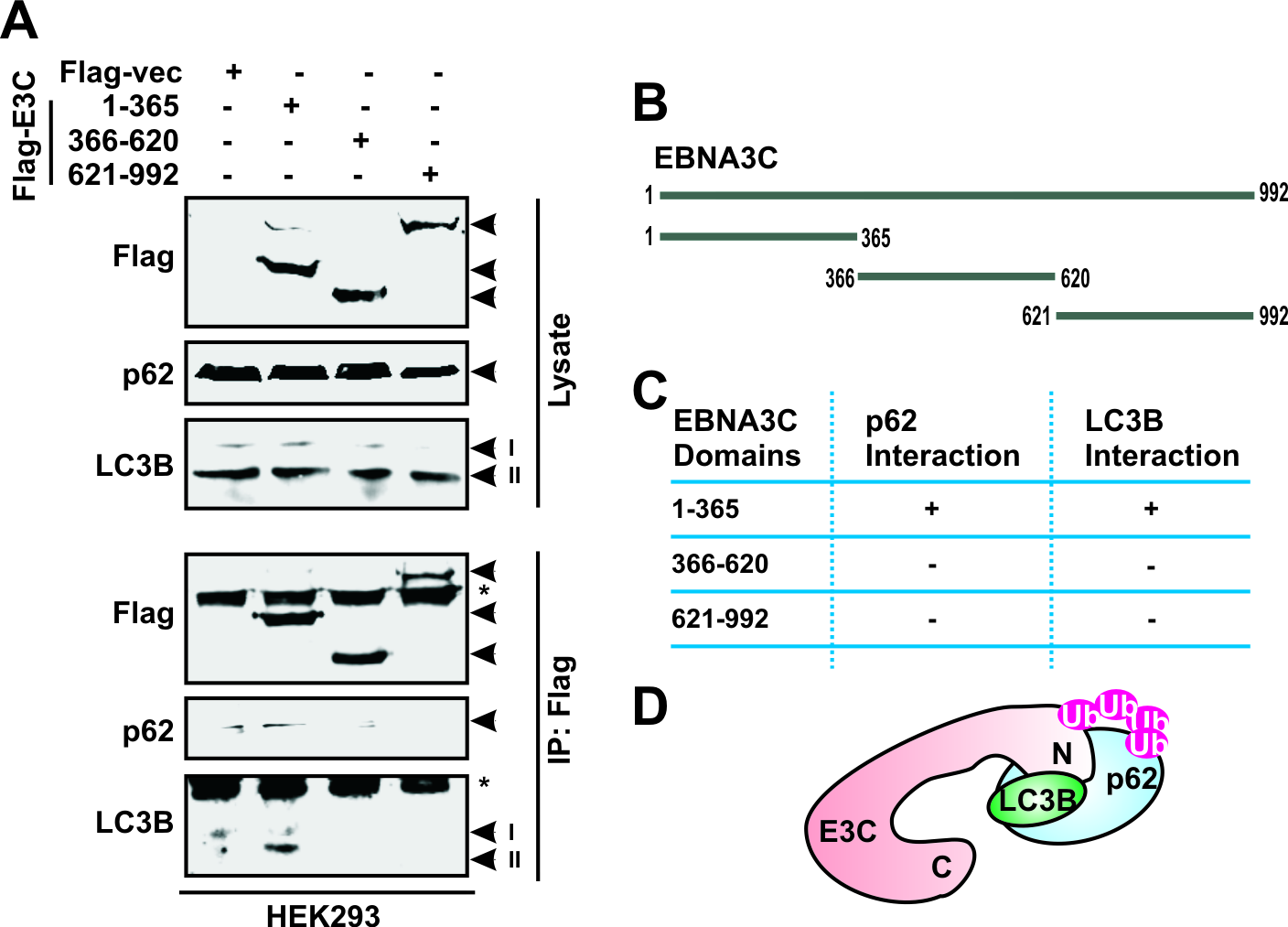

Supplement: S7 Fig — (A) HEK293 cells transiently transfected with expression plasmids for flag-tagged EBNA3C truncations (residues 1–365, 366–621 and 621–992) were subjected to co-immunoprecipitation study with anti-flag antibody. Western blots were performed with the indicated antibodies by stripping and reprobing the same membrane. * indicates IgG bands. (B) Schematic representation of EBNA3C truncations used in the co-immunoprecipitation experiment. (C-D) Summary and cartoon representation of the interaction study of different EBNA3C domains with p62 and LC3B. (TIF) [file ppat.1008105.s007.TIF]

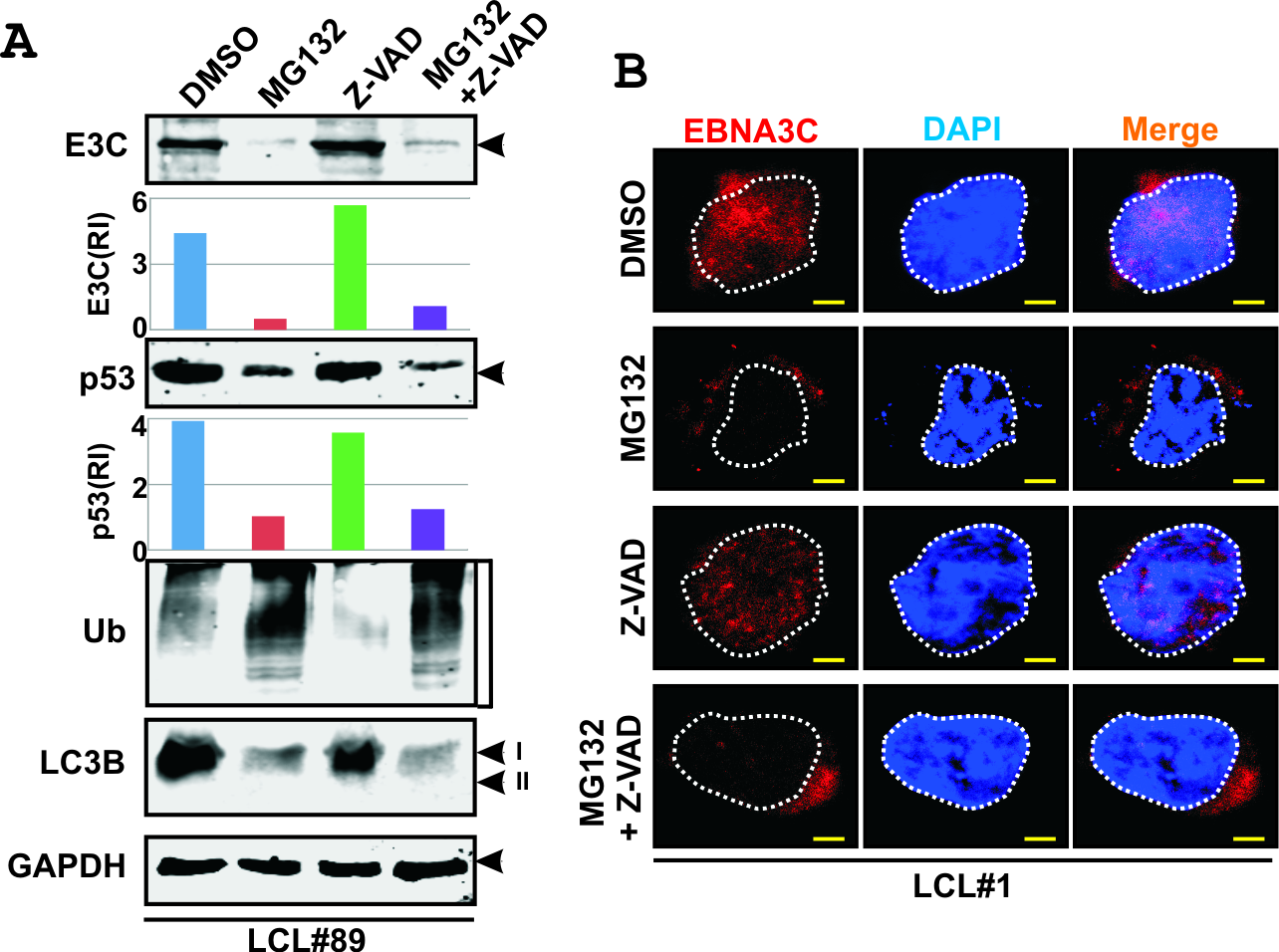

Supplement: S8 Fig — LCLs were either left untreated (DMSO control) or treated with 1 μM MG132, 50 μM pan-caspase inhibitor Z-VAD(OMe)-FMK (Z-VAD) or MG132 plus Z-VAD. 24 h post-treatment cells were subjected for either (A) western blot analysis or (B) immunostaining with the indicated antibodies. Each panel in (B) is representative picture of two independent experiments and nuclei were counterstained by DAPI before mounting the cells. Scale bars, 5 μm. In (A) GAPDH blot was used as loading control and protein bands were quantified by Odyssey imager software and represented as bar diagrams at the bottom of corresponding lanes. (TIF) [file ppat.1008105.s008.TIF]

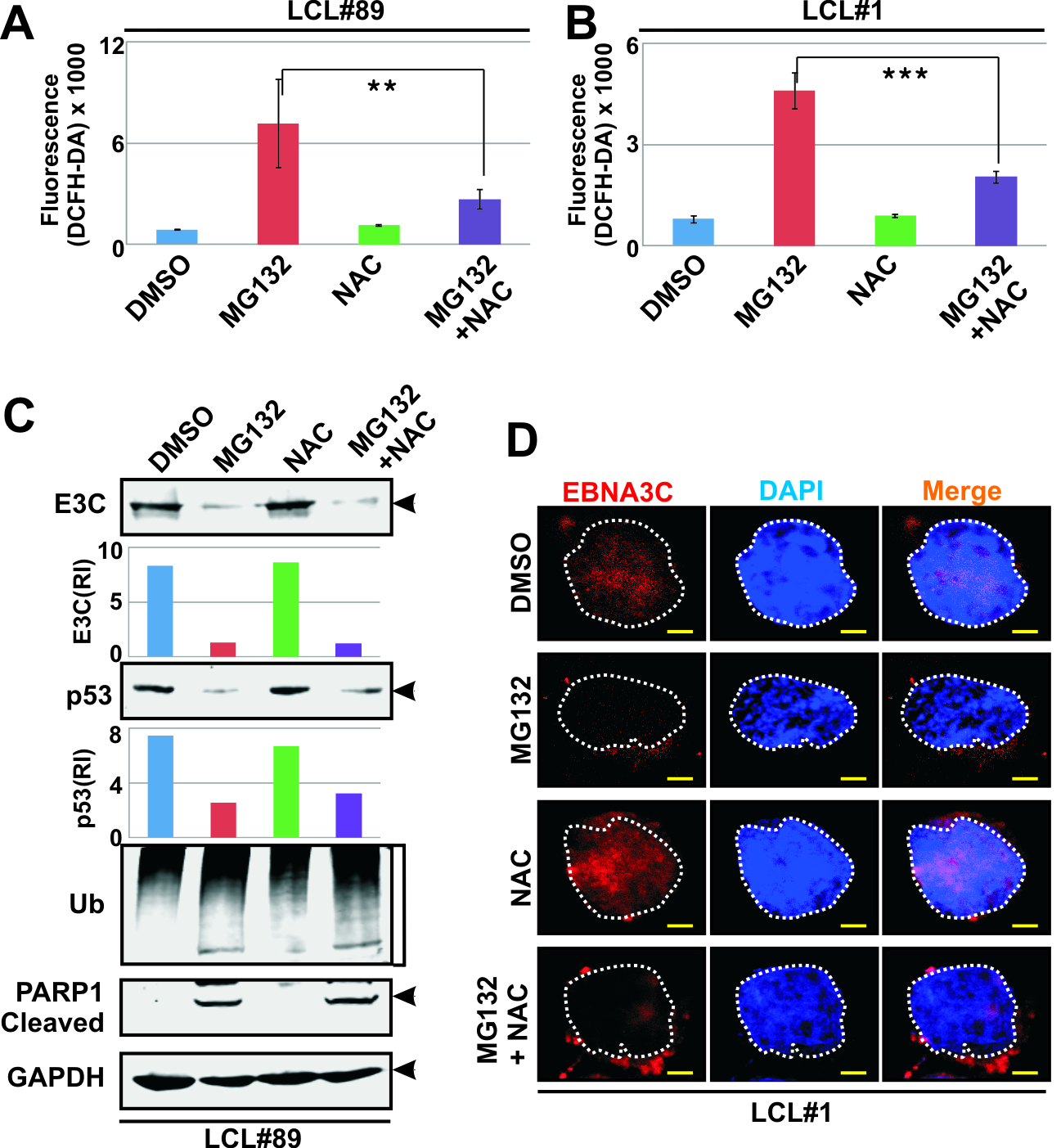

Supplement: S9 Fig — LCLs were either left untreated (DMSO control) or treated with 0.5 μM MG132, 1 mM N-Acetyl-L-cysteine (NAC) or MG132 plus NAC. 24 h post-treatment, cells were subjected for (A-B) ROS (Reactive Oxygen Species) measurement using DCFH-DA fluorescent probe, (C) western blot analysis and (D) immunostaining with the indicated antibodies. (C) GAPDH blot was used as loading control and protein bands were quantified by Odyssey imager software and represented as bar diagrams at the bottom of corresponding lanes. Each panel in (D) corresponds to single experiment of two independent experiments and nuclei were counterstained by DAPI before mounting the cells. Scale bars, 5 μm. (TIF) [file ppat.1008105.s009.TIF]

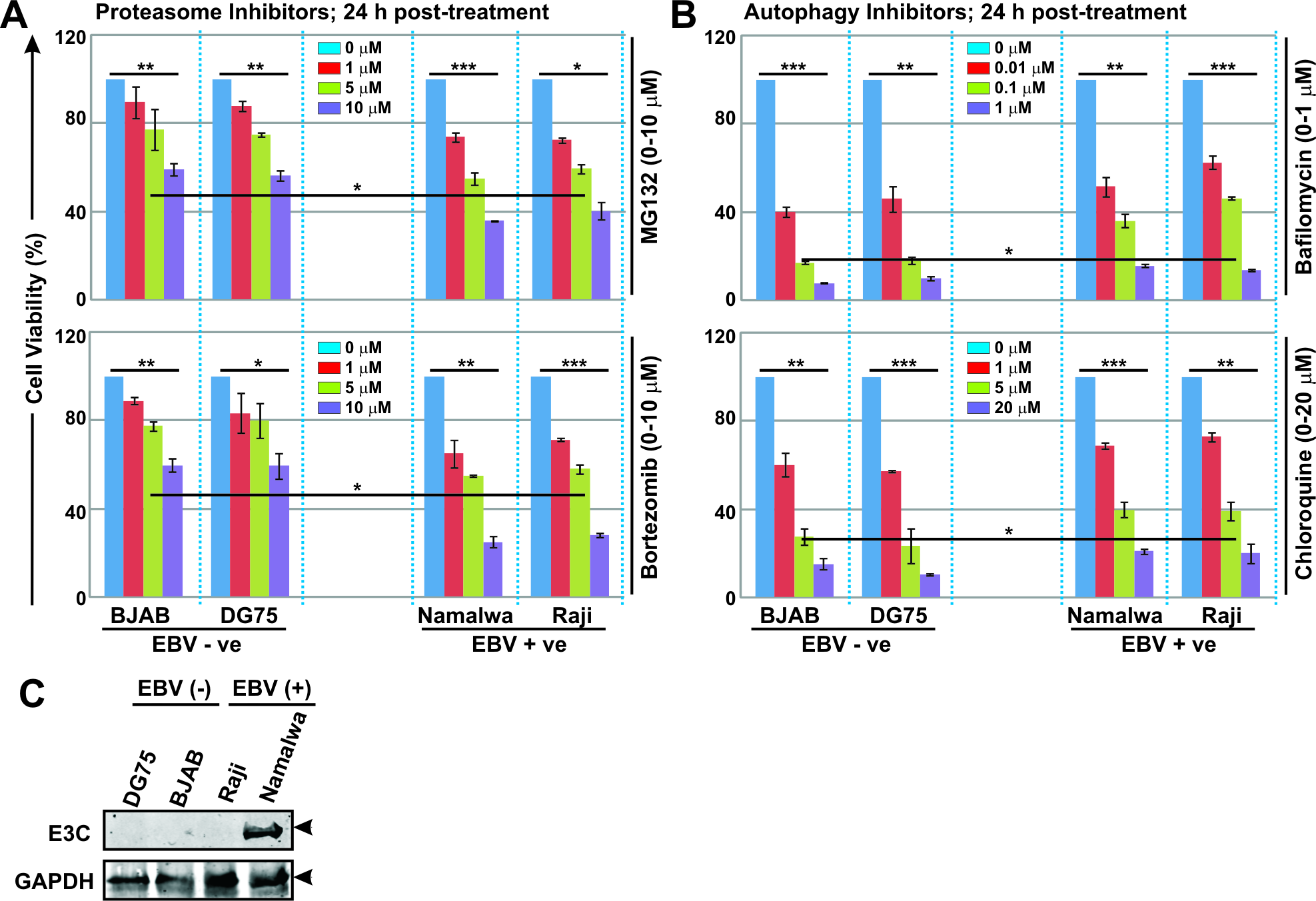

Supplement: S10 Fig — ~10 x 106 cells were harvested, lysed in RIPA buffer and subjected to western blot analyses with anti-GAPDH (as loading control) and anti-EBNA3C antibodies. (TIF) [file ppat.1008105.s010.TIF]

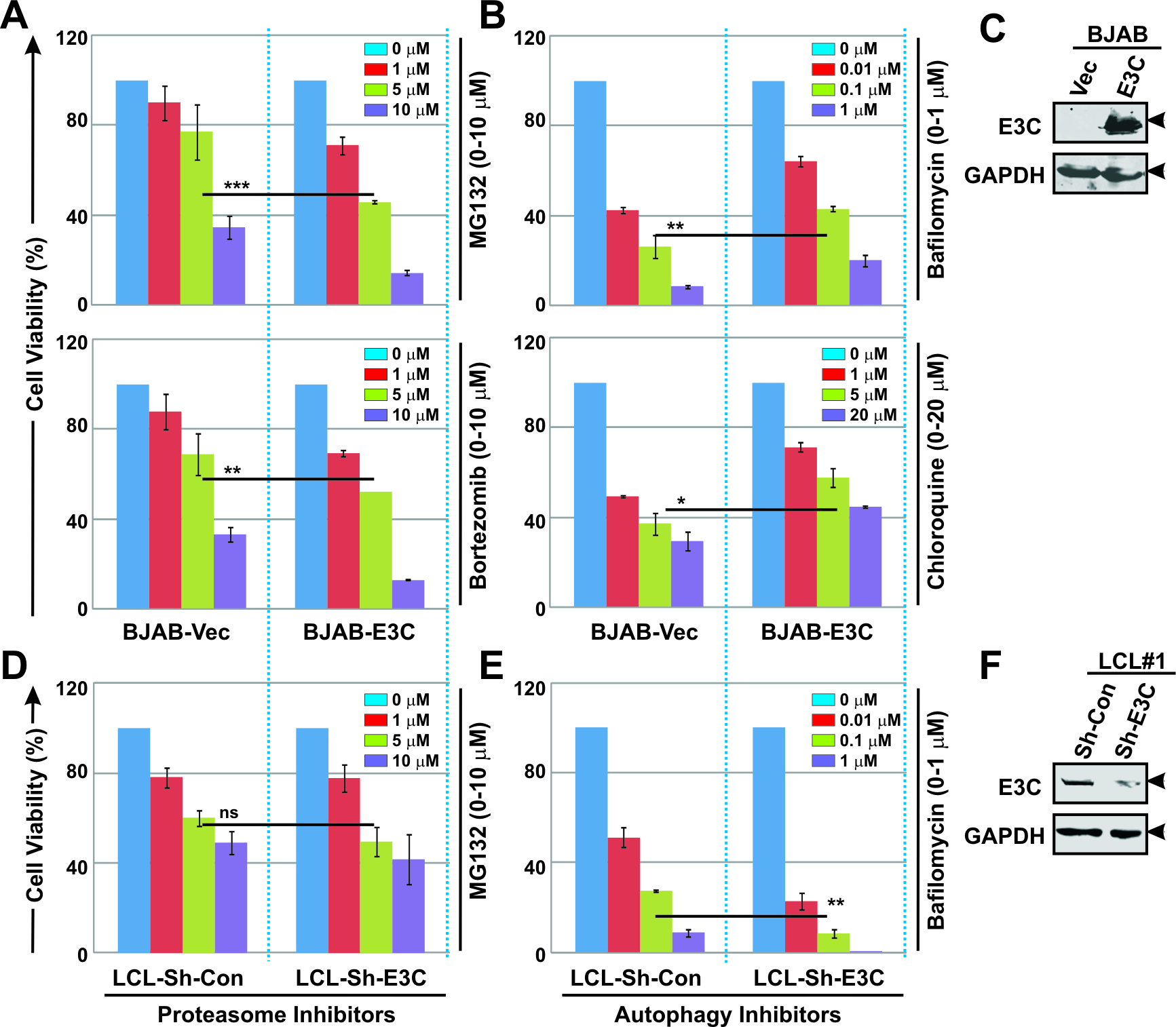

Supplement: S11 Fig — ~10 x 106 cells were harvested, lysed in RIPA buffer and subjected to western blot analyses with anti-GAPDH (as loading control) and anti-EBNA3C antibodies. (TIF) [file ppat.1008105.s011.TIF]
