# Supplementary material for: Proteasomal inhibition triggers viral oncoprotein degradation via autophagy-lysosomal pathway
Source: PLoS Pathog. 2020 Feb 24;16(2):e1008105. doi: 10.1371/journal.ppat.1008105 (PMC7058366; doi:10.1371/journal.ppat.1008105)
Supplement: S2 Table — Real-time PCR primers for both viral and cellular genes were designed from Primer-BLAST (https://www.ncbi.nlm.nih.gov/tools/primer-blast/). Reference Sequence ID (RefSeq ID) of each gene is mentioned. All primers were selected at annealing temperature of ~60°C. RefSeq ID of each gene is mentioned. (DOCX) [file ppat.1008105.s013.docx]

**Table S2.** **Real time PCR primers.**

| **SL No.** | **Gene Name** | **Reference Sequences** | **Primer Sequences** |
| --- | --- | --- | --- |
|  | EBNA3A | YP_401669.1 | Fw- 5’-GCCCTGGATGACAACATGGA-3’  Rv- 5’-CAGGTGGGCATCTTCTGCTT-3’ |
|  | EBNA3B | NC_007605.1 | Fw- 5’-CCCTTGCGGATGCAGCCAAT-3’  Fw- 5’-GGCTGATATGGAATGTGCCC-3’ |
|  | EBNA3C | YP_401671.1 | Fw- 5’-AAGGTGCATTTACCCCACTG-3’  Rv- 5’-AGCAGTAGCTTGGGAACACC-3’ |
|  | EBNA3A* | YP_401669.1 | Fw- 5’-CTAAGAACACTTCTTCAAGC-3’  Rv- 5’-CTCGGTATTTGAAATCTGG-3’ |
|  | EBNA3B* | NC_007605.1 | Fw- 5’-GGGATCTGAGCCTATTTCAC-3’  Fw- 5’-TTCCAACGCCTCTGCTTAAC-3’ |
|  | EBNA3C* | YP_401671.1 | Fw- 5’-GACATCACACCATATACCGC-3’  Rv- 5’-TGTTAGAAGCCAATGTCGCC-3’ |
|  | EBNA1 | YP_401677.1 | Fw- 5’-CATTGAGTCGTCTCCCCTTTGGAAT-3’  Rv- 5’-TCATAACAAGGTCCTTAATCGCATC-3’ |
|  | EBNA2 | YP_401644.1 | Fw- 5’-GAGACCAGAGCCAAACACCTCCAGT-3’  Rv- 5’-TTAGGGGTTGCCGTGTGTGAATTTC-3’ |
|  | LMP1 | YP_401722.1 | Fw- 5’-CCCGCACCCTCAACAAGCTACCGAT-3’  Rv- 5’-TTGTCAGGACCACCTCCAGGTGCGC-3’ |
|  | LMP2A | YP_401631.1 | Fw- 5’-CTCTCACTTCTACTCTTGGCAG-3’  Rv- 5’-AGTCAAACGGCGCCATCTCCTT-3’ |
|  | BALF1 | YP_401718 | Fw- 5’-GCTGGCCTTGAGGGCGCTGAGGACT-3’  Rv- 5’-CACCCACGGAAGCCCTCTGGACTTC-3’ |
|  | BCLF1 | YP_401697.1 | Fw- 5’-CCTCTTGGAATGCAGCTGGGGCCAG-3’  Rv- 5’-CCAATTATGACCTGCTGCGGCTGGA-3’ |
|  | BZLF1 | YP_401673.1 | Fw- 5’- CCAGGTTGAGGTGCTTCTCCCCCGG-3’  Rv- 5’-AACCGCTCCGACTGGGTCGTGGTTT-3’ |
|  | MAP1LC3B | NM_022818 | Fw- 5’-AAGCTGCTTCTCACCCTTGT-3’  Rv- 5’-GAGAAGACCTTCAAGCAGCG-3’ |
|  | SQSTM1 | NM_003900 | Fw- 5’-TTCTTTTCCCTCCGTGCTC-3’  Rv- 5’-GGATCCGAGTGTGAATTTCC-3’ |
|  | GABARAPL1 | NM_031412.4 | Fw- 5’-GGTGCATCATGAAGTTCCAG-3’  Rv- 5’-GGCTTTTGGAGCCTTCTCTA-3’ |
|  | CTSD | NM_001909.5 | Fw- 5’-AGCCCTCCAGCCTTCTG-3’  Rv- 5’-CGGATGGACGTGAACTTGT-3’ |
|  | HSPA8 | NM_006597.6 | Fw- 5’-TATTGGAGCCAGGCCTACAC-3’  Rv- 5’-AGTGGTTCGGTTTCCCTGAT-3’ |
|  | HSP90AA1 | NM_001017963.3 | Fw- 5’-GGTCCTGTGCGGTCACTTAG-3’  Rv- 5’-AAAGGCGAACGTCTCAACC-3’ |
|  | GAPDH | NM_001289745.3 | Fw- 5’-AATGAAGGGGTCATTGATGG-3’  Rv- 5’-AAGGTGAAGGTCGGAGTCAA-3’ |
|  | B2M | NM_004048.3 | Fw- 5’-GAGGCTATCCAGCGTACTCCA-3’  Rv- 5’-CGGCAGGCATACTCATCTTTT-3’ |
|  | RPLPO | NM_007475.5 | Fw- 5’-ATCTGCTTGGAGCCCACAT-3’  Rv- 5’-GCGACCTGGAAGTCCAACTA-3’ |

* primers for detecting intronic sequences in EBNA3 genes [53].
